# NineML: the network interchange for n**e**uroscience modeling language

**DOI:** 10.1186/1471-2202-12-S1-P330

**Published:** 2011-07-18

**Authors:** Ivan Raikov, Robert Cannon, Robert Clewley, Hugo Cornelis, Andrew Davison, Erik De Schutter, Mikael Djurfeldt, Padraig Gleeson, Anatoli Gorchetchnikov, Hans Ekkehard Plesser, Sean Hill, Mike Hines, Birgit Kriener, Yann Le Franc, Chung-Chan Lo, Abigail Morrison, Eilif Muller, Subhasis Ray, Lars Schwabe, Botond Szatmary

**Affiliations:** 1 Computational Neuroscience Unit, Okinawa Institute of Science and Technology, Okinawa, Japan; 2 University of Antwerp, Antwerp, Belgium

## 

The growing number of large-scale neuronal network models has created a need for standards and guidelines to ease model sharing and facilitate the replication of results across different simulators. To foster community efforts towards such standards, the International Neuroinformatics Coordinating Facility (INCF) has formed its Multiscale Modeling program, and has assembled a task force of simulator developers to propose a declarative computer language for descriptions of large-scale neuronal networks.

The name of the proposed language is "Network Interchange for Neuroscience Modeling Language" (NineML) and its initial focus is restricted to point neuron models.

The INCF Multiscale Modeling task force has identified the key concepts of network modeling to be 1) spiking neurons 2) synapses 3) populations of neurons and 4) connectivity patterns across populations of neurons. Accordingly, the definition of NineML includes a set of mathematical abstractions to represent these concepts.

NineML aims to provide tool support for explicit declarative definition of spiking neuronal network models both conceptually and mathematically in a simulator independent manner. In addition, NineML is designed to be self-consistent and highly flexible, allowing addition of new models and mathematical descriptions without modification of the previous structure and organization of the language. To achieve these goals, the language is being iteratively designed using several representative models with various levels of complexity as test cases.

The design of NineML is divided in two semantic layers: the Abstraction Layer, which consists of core mathematical concepts necessary to express neuronal and synaptic dynamics and network connectivity patterns, and the User Layer, which provides constructs to specify the instantiation of a network model in terms that are familiar to computational neuroscience modelers.

As part of the Abstraction Layer, NineML includes a flexible block diagram notation for describing spiking dynamics. The notation represents continuous and discrete variables, their evolution according to a set of rules such as a system of ordinary differential equations, and the conditions that induce a regime change, such as the transition from subthreshold mode to spiking and refractory modes.

The User Layer provides syntax for specifying the structure of the elements of a spiking neuronal network. This includes parameters for each of the individual elements (cells, synapses, inputs) and the grouping of these entities into networks. In addition, the user layer defines the syntax for supplying parameter values to abstract connectivity patterns.

The NineML specification is defined as an implementation-neutral object model representing all the concepts in the User and Abstraction Layers. Libraries for creating, manipulating, querying and serializing the NineML object model to a standard XML representation will be delivered for a variety of languages. The first priority of the task force is to deliver a publicly available Python implementation to support the wide range of simulators which provide a Python user interface (NEURON, NEST, Brian, MOOSE, GENESIS-3, PCSIM, PyNN, etc.). These libraries will allow simulator developers to quickly add support for NineML, and will thus catalyze the emergence of a broad software ecosystem supporting model definition interoperability around NineML.

